# DNA Induces Conformational Changes in a Recombinant Human Minichromosome Maintenance Complex*

**DOI:** 10.1074/jbc.M114.622738

**Published:** 2015-02-03

**Authors:** Emma L. Hesketh, Richard P. Parker-Manuel, Yuriy Chaban, Rabab Satti, Dawn Coverley, Elena V. Orlova, James P. J. Chong

**Affiliations:** From the ‡ Department of Biology, University of York, York YO10 5DD and; the § Department of Crystallography, Birkbeck College London, London WC1E 7HX, United Kingdom

**Keywords:** ATPase, DNA Helicase, DNA Replication, Electron Microscopy (EM), Recombinant Protein Expression, MCM, Minichromosome Maintenance

## Abstract

ATP-dependent DNA unwinding activity has been demonstrated for recombinant archaeal homohexameric minichromosome maintenance (MCM) complexes and their yeast heterohexameric counterparts, but in higher eukaryotes such as *Drosophila*, MCM-associated DNA helicase activity has been observed only in the context of a co-purified Cdc45-MCM-GINS complex. Here, we describe the production of the recombinant human MCM (hMCM) complex in *Escherichia coli*. This protein displays ATP hydrolysis activity and is capable of unwinding duplex DNA. Using single-particle asymmetric EM reconstruction, we demonstrate that recombinant hMCM forms a hexamer that undergoes a conformational change when bound to DNA. Recombinant hMCM produced without post-translational modifications is functional *in vitro* and provides an important tool for biochemical reconstitution of the human replicative helicase.

## Teaser

**Background:** The human minichromosome maintenance (hMCM) complex is an important component of the DNA replication apparatus.

**Results:** After being produced in *Escherichia coli*, hMCM has ATPase and DNA helicase activity and undergoes a conformational change when bound to DNA.

**Conclusion:** Recombinant hMCM is functional *in vitro*.

**Significance:** hMCM provides an important tool for the biochemical reconstitution of the human replicative helicase.

## Introduction

DNA replication is fundamental to the proliferation of all cells and, as such, has been the subject of intense scrutiny over many years. Although this work has demonstrated many unifying similarities among all eukaryotes with regard to DNA replication, it has also become obvious that there are a number of differences in the details regarding these systems. Significantly, one of the main model organisms for eukaryotic DNA replication, the yeast *Saccharomyces cerevisiae*, has a closed mitosis that requires replication proteins to cross the nuclear envelope in a manner not required in other eukaryotes (1). The minichromosome maintenance (MCM)^7^ proteins are a good example of such proteins, and *S. cerevisiae* MCM proteins possess insertions to cater to this requirement (Table 1). The models used to study replication in *Xenopus* and *Drosophila* are egg-based and contain unusually high levels of (likely preassembled) replication complexes not normally seen in post-embryonic systems (2). Data from human tissue culture studies are based mainly on transformed cells, which probably do not accurately reflect the cell cycle controls in primary explants (3). Thus, the use of recombinant components, which may more accurately reconstitute robust DNA helicase activity biochemically, is of significant interest in understanding human DNA replication processes.

**TABLE 1:** **Comparison of *S. cerevisiae* and *H. sapiens* MCM amino acid insertions** aa, amino acids.

MCM	*S. cerevisiae* (aa)	*H. sapiens* (aa)	aa difference (*S. cerevisiae* − *H. sapiens*)	Insertion locations
2	868	904	−36 (−4%)	C
3	971	808	163 (20%)	N, C
4	933	863	70 (8%)	N
5	775	734	41 (6%)	N
6	1017	821	196 (24%)	N, C
7	845	719	126 (18%)	N

The MCM complex is essential for DNA replication in eukaryotes and archaea, where it is believed to act as the replicative DNA helicase (reviewed in Ref. 4). Homohexameric archaeal MCM complexes have provided useful models for studying DNA helicase activity (5, 6). The production of a number of crystal structures has facilitated mapping of key enzyme residues and provided insight into the molecular mechanisms utilized in DNA unwinding and translocation (7, 8). In addition to its essential role in DNA replication, the heterohexameric eukaryotic MCM2–7 complex has been implicated in DNA damage responses, checkpoint signaling, transcription, and chromatin remodeling (9–12).

Isolation of intact functional MCM heterohexamers has proved challenging. Functional MCM complexes purified from *Xenopus* egg extracts using a replication licensing factor assay demonstrated an additional requirement for Cdt1 in MCM chromatin loading (13, 14). A Cdc45-MCM-GINS complex with DNA helicase activity has been isolated from *Drosophila* egg extracts (15). MCM function is post-translationally modulated *in vivo* by phosphorylation, although this is not essential for replication elongation (16). The presence of inhibitory phosphorylations might explain why some purified MCM complexes produced in eukaryotic expression systems do not demonstrate significant helicase activity. Current methods for MCM2–7 complex analysis rely on purification from yeast or insect cells, potentially complicating the interpretation of results, as the purified MCM2–7 complexes may have been post-translationally modified prior to purification.

We report the development of a bacterial expression protocol that allows the production of a recombinant human MCM2–7 (hMCM) complex in *Escherichia coli*. Our hMCM complex exhibits DNA unwinding and ATP hydrolysis with a different salt sensitivity from that reported for the yeast complex (17). Structures of the protein obtained by EM methods show that hMCM forms an asymmetric hexameric complex that undergoes a conformational change in the presence of a forked DNA substrate that it is also capable of unwinding. The generation of recombinant hMCM represents the development of an important tool for understanding the mechanisms governing human DNA replication. This may ultimately improve our ability to manipulate cell proliferation and therefore design useful and specific cancer treatments.

## EXPERIMENTAL PROCEDURES

### 

#### 

##### Expression and Purification of the MCM2–7 Complex

PCR-amplified MCM2–7 cDNAs (see primers in Table 2) were cloned into Ek/LIC Duet vectors (Novagen). pET32-MCM2/7, pRSF-MCM3/5, and pCDF-MCM4/6 were transformed into *E. coli* Rosetta 2(DE3) cells (Novagen) and grown in a 75-liter fermenter. The hMCM complex was bound to HIS-Select cobalt affinity gel (Sigma) and passed over Superdex 200 gel filtration and Mono Q columns. Fractions containing MCM2–7 were pooled; dialyzed against 25 mm HEPES (pH 8.0), 200 mm sodium glutamate, 1 mm DTT, and 0.5 mm PMSF; flash-frozen in small aliquots; and stored at −80 °C. Site-directed mutagenesis of pET32-MCM2/7, pRSF-MCM3/5, and pCDF-MCM4/6 produced inactive point mutations in the Walker A motif for each hMCM subunit (MCM2(K529E), MCM3(K351E), MCM4(K516E), MCM5(K387E), MCM6(K402E), and MCM7(K387E)). ATPase-deficient mutant protein was produced and purified as described for the WT type protein.

**TABLE 2:** **List of oligonucleotides used in this study**

Oligonucleotide	Sequence
MCM2	
Forward	gcgggcccggcctccatggcggaatcatcggaatccttcacc
Reverse	gaggagaagcccggtcagaactgctgcaggatcattttcc
MCM3	
Forward	gcgggcccggccttcatggcgggtaccgtggtgctggac
Reverse	gaggagaagcccggtcagatgaggaagatgatgccctcag
MCM4	
Forward	gcgggcccggccttcatgtcgtccccggcgtcgacccc
Reverse	gaggagaagcccggtcagagcaagcgcacggtcttccc
MCM5	
Forward	gacgacgacaagatgtcgggattcgacgatcctggc
Reverse	cgcgggcggccgtcacttgaggcggtagagaaccttgc
MCM6	
Forward	gacgacgacaagatggacctcgcggcggcagcgg
Reverse	cgcgggcggccgtcaatcttcgagcaagtagttaggg
MCM7	
Forward	gacgacgacaagatggcactgaaggactacgcgctag
Reverse	cgcgggcggccgtcagacaaaagtgatccgtgtccggg
HF150	cctggcgttacccaacttaatcgccttgcagcacatccccctttctttttttttttttttttttttttttttttttttttttttttttttttttttttttttttt
HR80	tttttttttttttttttttttttttttttttttttgaaagggggatgtgctgcaaggcgattaagttgggtaacgccagg
HS1	gggacgcgtcggcctggcacgtcggccgctgcggccaggcacccgatggcgtttgtttgtttgtttgtttgttt
HS2	tttgtttgtttgtttgtttgtttgtttgtttgccgacgtgccaggccgacgcgtccc

##### Western Blotting

Individual MCM subunits were confirmed by Western blotting the purified protein using primary antibodies: anti-MCM2 (CS732) (18), anti-MCM3 (clone 3A2; Medical & Biological Laboratories), anti-MCM4 (G-7; Santa Cruz Biotechnology), anti-MCM5 (clone 33; BD Biosciences), anti-MCM6 (clone 1/MCM6; BD Biosciences), and anti-MCM7 (141.2; Santa Cruz Biotechnology).

##### ATPase Assays

Reactions were modified from Ref. 6 as follows. Reactions containing 30 mm K_2_HPO_4_/KH_2_PO_4_ buffer (pH 8.5), 1 mm DTT, 100 μg/ml BSA, 2% (v/v) glycerol, 10 mm magnesium acetate, 1.5 nmol of unlabeled ATP, 3.08 pmol of [α-^32^P]ATP (800 Ci/mmol; ICN), 3.5 nm circular dsDNA (pUC119 unless stated otherwise), and 176 nm protein were assembled on ice.

##### DNA Helicase Substrate

Oligonucleotide HS2 (Table 2) was radiolabeled and annealed to oligonucleotide HS1 as described (6).

##### Helicase Assays

Helicase reactions (4 nm ^32^P-labeled forked substrate, MCM2–7 as indicated, 30 mm K_2_HPO_4_/KH_2_PO_4_ buffer (pH 8.5), 300 mm potassium glutamate, 1 mm DTT, 100 μg/ml BSA, 2% (v/v) glycerol, 10 mm magnesium acetate, and 4 mm ATP) were incubated for 1 h at 37 °C and stopped using 0.25 volume of 80 mm EDTA, 0.8% (w/v) SDS, 40% (v/v) glycerol, 0.04% (w/v) xylene cyanol, and 0.04% (w/v) bromphenol blue. Reaction products were separated on Tris borate/EDTA-11% (w/v) polyacrylamide gels (2 h, 80 V). Gels were dried, imaged, and quantified using a Bio-Rad Molecular Imager FX and Quantity One software.

##### Binding of Duplex DNA to hMCM for EM

No-salt annealing buffer (200 mm HEPES (pH 8.0) and 5 mm EDTA) was added to 1 μm HF150 (150 bp) and 1 μm HR80 (80 bp) (Table 2). DNA was annealed at 95 °C for 3 min, followed by cooling at 0.02 °C/s to 23 °C. The temperature was kept at 23 °C for 1 min. hMCM (8 μg) was incubated with 60 nm duplex DNA in annealing buffer (50 mm HEPES (pH 7.5), 2 mm DTT, 50 μg/ml BSA, 10 mm magnesium acetate, and 4 mm ATP) for 60 min at 37 °C to bind hMCM and duplex DNA. Samples were snap-frozen in liquid nitrogen.

##### Electron Microscopy

hMCM samples were applied to continuous carbon grids and stained with freshly made methylamine tungstate (pH 7). Data were collected on an FEI T12 microscope at a magnification of ×67,000 and an accelerating voltage of 120 kV, recorded on Kodak SO-163 films, and digitized using a Zeiss Photoscan densitometer (14-μm scanning step, corresponding to 2.5 Å/pixel) before analysis.

##### Image Processing

Particle picking was carried out automatically using Boxer (EMAN suite (19)). Analysis of the contrast transfer function and correction was completed using CTFIT (EMAN suite (19)). Image analysis was performed with IMAGIC-5 (20). Images were normalized to the same S.D. and bandpass-filtered. The low-resolution cutoff was ∼100 Å to remove uneven background in particle images, and the high-resolution cutoff was ∼7 Å. Images were subjected to an alignment procedure, followed by statistical analysis. Alignment and classification of images were performed as described previously (20) and yielded classes representing characteristic views of the molecule. Primary structural analysis of hMCM and hMCM plus DNA complexes was performed using an *ab initio* approach in which the orientations of the best 10–15 image classes were determined by angular reconstitution using C1 startup. Three-dimensional maps were calculated using the exact filtered backprojection algorithm (20). Structural analysis was performed using several starting models with several different sets of image classes for *ab initio* reconstructions. The first reconstructions were used for the following rounds of alignment and classification of images. The structures of the complexes were refined by an iterative procedure with the number of classes gradually increased. The final reconstruction for hMCM alone was calculated from the best 100 classes containing ∼11 images each. For hMCM plus DNA, the final reconstruction was calculated from the best 155 classes containing ∼10 images each. Resolution of the map was assessed using the 0.5 threshold of Fourier shell correlation (21), which corresponds to 23 Å. Domain fitting into the three-dimensional map of hMCM and hMCM plus DNA complexes was performed manually with UCSF Chimera (22). Illustrations were generated using UCSF Chimera. Surface representations (unless stated otherwise) are displayed at a threshold level of 3σ (S.D. of densities within EM maps), which corresponds to ∼100% of the expected mass at a specific protein density of 0.84 kDa/Å^3^.

## RESULTS

### 

#### 

##### Production of a Soluble hMCM Complex

**FIGURE 1.**
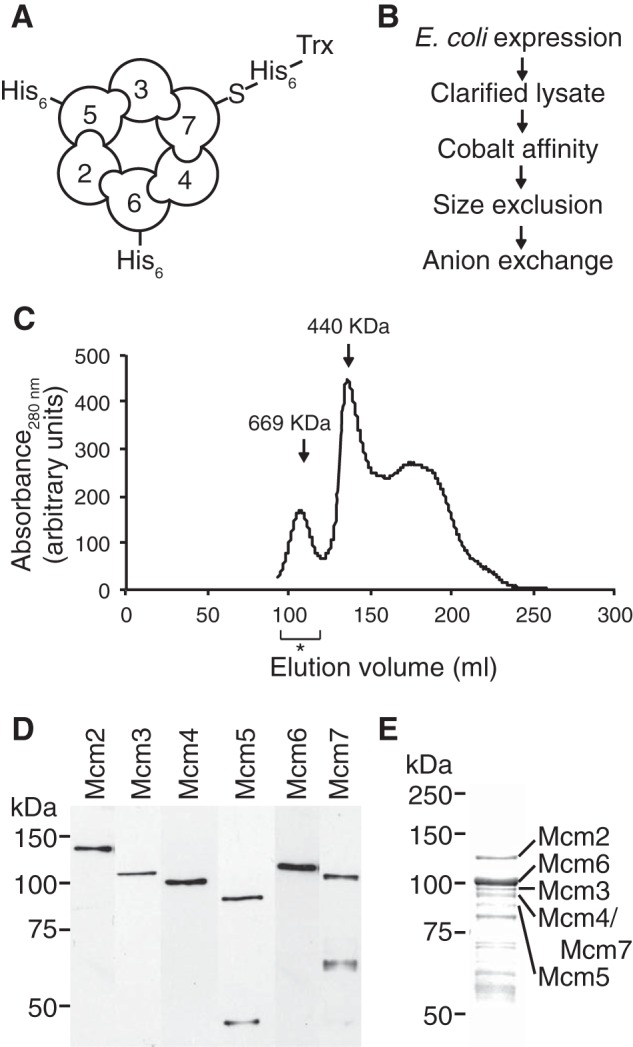
**Purification of a bacterially expressed hMCM complex.** *A*, schematic of the recombinant hMCM complex. Shown is the predicted arrangement of MCM2–7 subunits indicating which subunits have N-terminal affinity tags to aid in protein purification. *B*, purification scheme for the recombinant hMCM complex. *C*, gel filtration elution profile for the recombinant hMCM complex. The elution fractions pooled are indicated by the *asterisk*. The protein markers thyroglobulin (669 kDa) and ferritin (440 kDa) are shown. *D*, Western blot using antibodies specific to individual MCM subunits, showing all six hMCM subunits in the purified complex. *E*, the purified hMCM complex separated by 10% (w/v) SDS-PAGE and visualized by Coomassie Blue staining.

To avoid potential activity-inhibiting phosphorylation by kinases present in eukaryotic expression systems, we coexpressed the hMCM2–7 proteins as a stable soluble complex in *E. coli* (Fig. 1*A*). The complex was purified according to the scheme outlined in Fig. 1*B*. The gel filtration elution profile for hMCM shows that the peak purified is at ∼600 kDa (Fig. 1*C*). The presence of all six hMCM subunits in the purified complex was demonstrated by Western blotting using specific antibodies (Fig. 1*D*). Consistent with our Western blot results, visualization of the complex using Coomassie Blue-stained SDS-polyacrylamide gel revealed the presence of some degradation products (confirmed by MS analysis) in addition to all six full-length hMCM subunits (Fig. 1*E*). The MCM6 band is more intense than the bands of the other subunits in the Coomassie Blue gel (Fig. 1*E*), which could be due to differential staining based on protein composition or a slight excess of uncomplexed MCM6 being present in the soluble fraction after expression in *E. coli*. Typically, we produce 82.5 μg of hMCM/liter of *E. coli* culture. A complex harboring inactivating point mutations in the Walker A motifs of each hMCM subunit (MCM2(K529E), Mcm3(K351E), MCM4(K516E), MCM5(K387E), MCM6(K402E), and MCM7(K387E)) was produced in the same way.

##### ATP Hydrolysis Activity of hMCM

**FIGURE 2.**
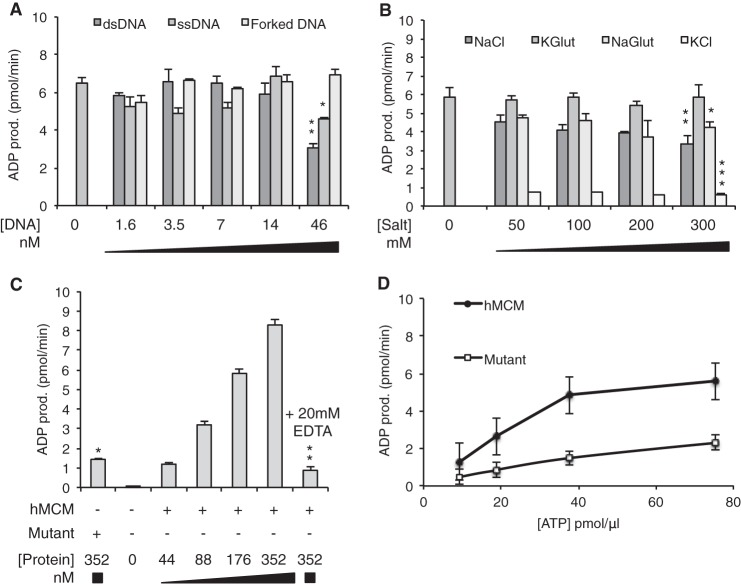
**WT hMCM possesses ATPase activity.** *A*, 46 nm closed circular ssDNA (hMCM hexamer/DNA molar ratio of 3.8:1) reduced ATP hydrolysis by one-third, and closed circular dsDNA reduced ATP hydrolysis by ∼50%. Forked DNA had little overall effect. Statistics compare the *labeled bars* with 0 nm DNA. *, *p* = 0.022; **, *p* = 0.0019. *B*, potassium glutamate (*KGlut*) had no effect on ATPase activity in the presence of 3.5 nm dsDNA. Increasing the concentrations of NaCl and sodium glutamate (*NaGlut*) reduced ATP hydrolysis, and the addition of KCl greatly reduced ATP hydrolysis. Statistics compare *labeled bars* with 0 mm salt. *, *p* = 0.018; **, *p* = 0.013; ***, *p* = 0.00027. *C*, ATPase activity increases with protein concentration in the presence of 3.5 nm dsDNA and is inhibited by the addition of 20 mm EDTA to chelate the Mg^2+^. ATPase-deficient mutant hMCM showed 6-fold lower ATP hydrolysis activity compared with WT hMCM. Statistics compare *labeled bars* with 352 nm hMCM. *, *p* = 0.0015; **, *p* = 0.00014. *D*, rate of ATP hydrolysis by WT hMCM and ATPase-deficient mutant hMCM in molecules of ADP released per min with optimized conditions (3.5 nm dsDNA, 300 mm potassium glutamate, and 176 nm hMCM). The maximum rate of ATP hydrolysis was 16.7 pmol of ADP produced (*prod.*) per min/pmol of WT hMCM and 3.9 pmol of ADP produced per min/pmol of mutant hMCM in the presence of 3.5 nm dsDNA. The data in *A–C* are mean values of three replicates, and the data in *D* are mean values of two assays. *Error bars* show S.D.

The purified WT hMCM complex and ATPase-deficient mutant complexes were tested for their ability to hydrolyze ATP in the presence and absence of a series of DNA substrates (at hMCM hexamer/DNA molar ratios ranging from 110:1 to 3.8:1) (Fig. 2*A*). hMCM exhibited ATP hydrolysis, which was not increased by the addition of DNA. High concentrations (46 nm) of closed circular dsDNA inhibited ATP hydrolysis by ∼50% compared with lower concentrations of dsDNA, possibly due to a substrate competition effect preventing the MCM proteins from forming a productive complex. A similar but smaller effect was observed for closed circular ssDNA. A forked DNA substrate had a negligible effect on hydrolysis activity at the concentrations tested. Subsequent ATP hydrolysis assays were carried out in the presence of 3.5 nm dsDNA.

On the basis of previous reports of specific salt requirements for yeast MCM activity *in vitro* (17), we examined the ability of hMCM to hydrolyze ATP in the presence of sodium chloride, sodium glutamate, potassium chloride, and potassium glutamate (Fig. 2*B*). Increased sodium chloride concentrations resulted in a statistically significant decrease in ATP hydrolysis. A similar effect was observed for sodium glutamate. The addition of 50 mm potassium chloride resulted in a pronounced inhibition of ATPase activity. Strikingly, the presence of 300 mm potassium glutamate, twice the physiological salt concentration, had no effect on ATPase activity (Fig. 2*B*). As expected, increasing concentrations of hMCM resulted in increased ATP hydrolysis (Fig. 2*C*). The addition of 20 mm EDTA significantly reduced ATP hydrolysis by hMCM, as did replacing the WT protein with the ATPase-deficient mutant complex (Fig. 2*C*), as expected from previous studies in *S. cerevisiae* (5, 23). Using the optimum assay conditions identified (3.5 nm dsDNA, 300 mm potassium glutamate, and 176 nm hMCM), we measured the rate of ATP hydrolysis for WT and mutant hMCM (Fig. 2*D*). The maximum rate of ATP hydrolysis was 16.7 pmol of ADP released per min/pmol of WT hMCM compared with 3.9 pmol of ADP released per min/pmol of mutant hMCM.

##### ATP-dependent DNA Unwinding by hMCM

**FIGURE 3.**
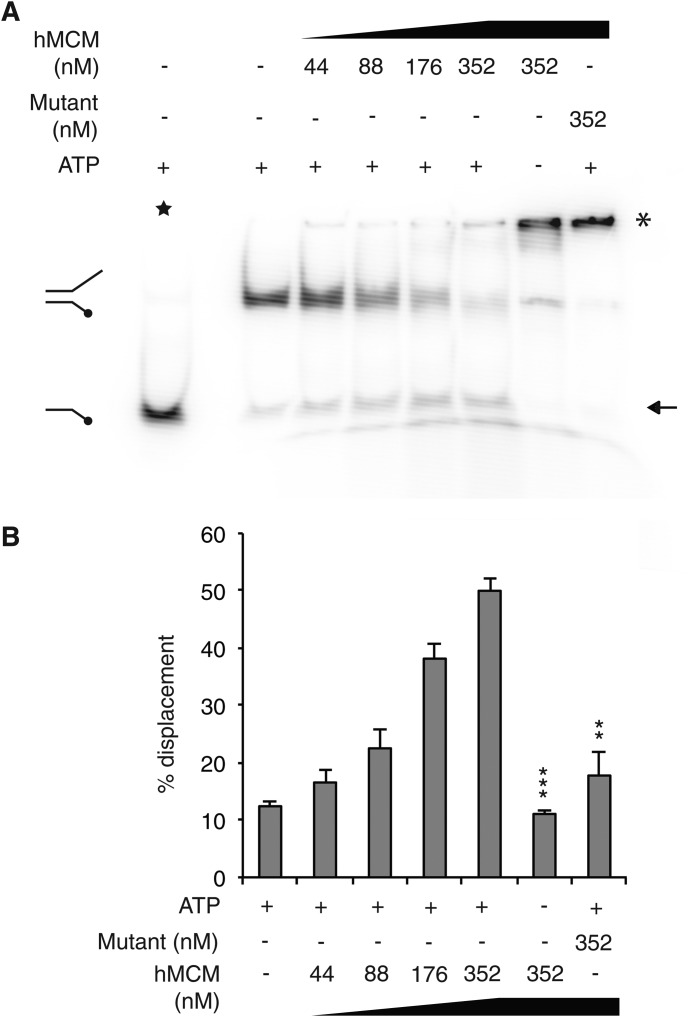
**Recombinant hMCM displays DNA helicase activity.** *A*, a forked DNA substrate was incubated with increasing concentrations of hMCM in the absence or presence of ATP. Heat-denatured boiled substrate (★) and no-protein lanes were included as controls. The *arrow* indicates the position of displaced substrate, and the *asterisk* indicates substrate with unusual mobility, perhaps indicating that hMCM that is bound to DNA. *B*, the amount of single-stranded substrate in each reaction was quantified as a percentage of the boiled substrate control. The data shown are mean values for four independent assays, an example of which is shown in *A. Error bars* indicate S.E. Statistics compare *labeled lanes* with 352 nm hMCM plus ATP. ***, *p* = 0.00013; **, *p* = 0.0014.

We tested our purified recombinant hMCM complexes for DNA helicase activity using a forked DNA substrate in the presence or absence of ATP. WT hMCM exhibited a protein concentration- and ATP-dependent DNA unwinding activity (Fig. 3). With WT hMCM, we observed 38 and 50% displacement by 176 m and 352 nm hMCM2–7, respectively (Fig. 3), broadly comparable with the reported 52% displacement by 110 nm yeast Mcm2–7 (17). Under conditions in which ATP could not be hydrolyzed, *i.e.* either WT hMCM in the absence of ATP or mutant hMCM in the presence of ATP, helicase activity was substantially reduced (Fig. 3*B*), consistent with ATP hydrolysis being required for helicase activity.

##### EM Structure of Recombinant hMCM

**FIGURE 4.**
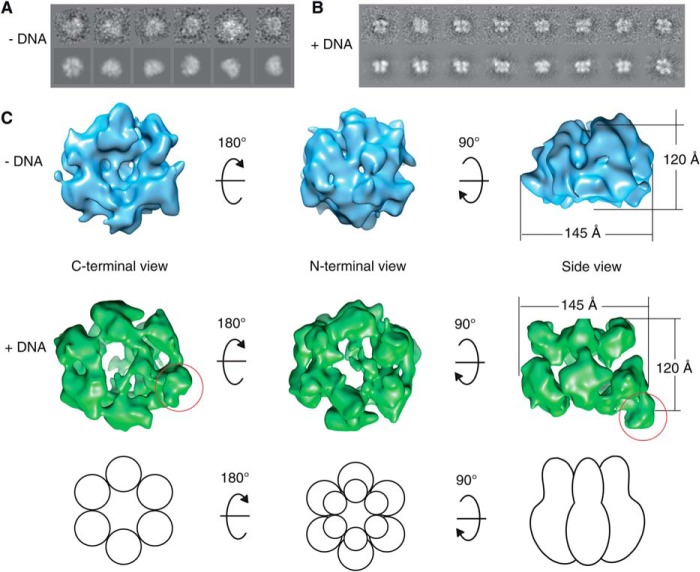
*A*, hMCM classes and reprojections used in the final reconstruction for hMCM alone. *B*, same as in *A* for hMCM plus DNA. *C*, negative stain, single-particle asymmetric EM three-dimensional reconstruction of hMCM to 23 Å resolution. *Upper row*, hMCM alone (*blue*) from three different aspects. The complex undergoes a conformational change when hMCM is bound to forked DNA (*middle row*, *green*). The *red circle* highlights protrusion thought to be DNA binding to hMCM. Sizes are indicated in angstroms. *Lower row*, schematic representation of hMCM subunit configuration.

**FIGURE 5.**
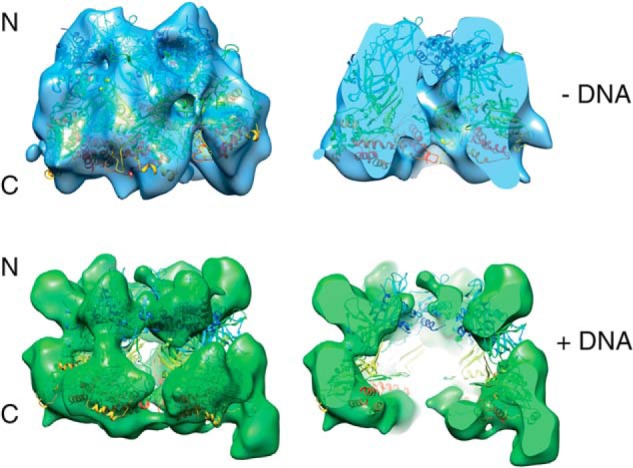
**The crystal structure of *S. solfataricus* MCM fits into our reconstructions.** Shown is hMCM alone (*blue*) and bound to forked DNA (*green*) fitted with the nearly full-length *S. solfataricus* MCM crystal structure (Protein Data Bank ID 3F9V) (8). The full-length hMCM structure (*left*) and a slice through hMCM (*right*) show a central cavity in both reconstructions.

The structures of the purified hMCM complex alone and bound to forked DNA were obtained by EM of negatively stained particles. Structures were obtained using a single-particle approach with C1 symmetry (Fig. 4). Our results reveal that the complex forms ring-shaped hexamers with a diameter of 145 Å and a height of 120 Å (Fig. 4*C*). Our asymmetric reconstructions (Fig. 4) clearly show that the complexes contain six subunits. However, the resolution does not allow us to identify the position of each subunit. When observed in the presence of forked DNA (Fig. 4*C*, *lower row*), the hMCM structure has a different conformation, with a more defined two-tiered hexameric shape and a more obviously open central cavity. The atomic model of the hexamer of *Sulfolobus solfataricus* MCM (Protein Data Bank ID 3F9V) fits neatly into our reconstruction (Fig. 5).

## DISCUSSION

ATP hydrolysis has been demonstrated for MCM complexes derived from archaea (5) and eukaryotes (23, 24). We found that the addition of DNA did not stimulate ATP hydrolysis by purified hMCM. This is consistent with reports of *S. cerevisiae* Mcm2–7, in which DNA did not stimulate ATP hydrolysis (23, 25). It is worth noting that this is in contrast to the Mcm4-Mcm6-Mcm7 subcomplex, which is stimulated by DNA (26, 27). In agreement with observations from archaeal MCM proteins (5), high concentrations of dsDNA were in fact inhibitory. With respect to salt sensitivity, 50 mm potassium chloride strongly inhibited the ATPase activity of hMCM, whereas potassium glutamate, even at a concentration of 300 mm, had no such effect. These findings are consistent with results reported for the DNA helicase activity of *S. cerevisiae* MCM (17).

Binding and hydrolysis of ATP by MCM purified from *S. cerevisiae* have recently been shown to be required for Cdt1 release and double-hexamer formation (28), with the ATPase sites of different MCM subunits implicated in different stages of MCM loading and activation (29). The interface between subunits 2 and 5 of the *S. cerevisiae* Mcm2–7 complex is thought to function as an ATP-dependent “gate,” the opening of which enables the toroidal complex to be loaded onto topologically closed DNA (17). The recombinant hMCM complex produced here is an important tool to analyze the loading and activation of the human complex in light of this finding.

Robust ATP-dependent DNA helicase activities were first demonstrated using archaeal homohexamers (5). Limited DNA helicase activity was originally demonstrated for a hexameric complex purified from HeLa cells that contained hMCM subunits 4, 6, and 7, probably as a dimer of trimers (24). This activity was inhibited by the addition of mouse MCM2 (30). The salt-sensitive DNA helicase activity of a heterohexameric Mcm2–7 complex was first demonstrated using *S. cerevisiae* proteins purified after baculovirus expression (17), and DNA unwinding activity of higher eukaryotic MCM complexes has been observed with the Cdc45-MCM-GINS complex from *Drosophila* (15). Here, we described the first demonstration of helicase activity for the hMCM2–7 complex.

Our results show that ATP hydrolysis is a requirement for DNA helicase activity. The negligible unwinding and ATPase activities in the mutant hMCM assays suggest that the mutant and WT protein preparations were both free from contaminating *E. coli* helicases/ATPases. Under ATPase null conditions (either no ATP or mutant hMCM), a large proportion of the helicase substrate migrated more slowly on native polyacrylamide gel. Similar mobility shift effects were observed previously for the *Methanothermobacter thermautotrophicus* MCM complex when samples were incubated on ice (6). This suggests that the hMCM protein binds to the substrate in the absence of ATP (or ATP hydrolysis) but cannot unwind it. This is consistent with the idea that ATP hydrolysis is not required for DNA-protein interactions but is required for DNA unwinding (31).

Overall, these results indicate that the recombinant hMCM complex exhibits DNA helicase activity and that post-translational modifications to hMCM or accessory proteins such as Cdc45 and GINS are not required for the unwinding of naked DNA. The presence of Cdc45 and GINS may be required only for remodeling or unwinding DNA packaged into chromatin.

The size and shape of our hMCM complex are consistent with the organization of oligomeric complexes reported for MCM from other eukaryotes (32, 33). Analysis of a population of *Drosophila* Mcm2–7 complexes revealed that they exist in two different states: a planar notched ring and an open spiral shape (32). Reconstructions of Mcm2–7 from *Encephalitozoon cuniculi* suggest that this Mcm2–7 complex is naturally found in the open spiral shape (34). Our reconstruction of the human complex is more similar to the notched ring, similar to *S. cerevisiae* (35), but this does not preclude the existence of a minority of spiral-shaped complexes in our sample.

Interestingly, the conformation taken by hMCM in the presence of DNA is somewhat similar to that which has been reported for *S. cerevisiae* Mcm2–7 in the absence of DNA (36). One possible reason for the differences observed between the yeast and human proteins in the absence of DNA could be the differences in their primary sequences (outlined in Table 1). The prominent projection that appears on the C-terminal surface of one of the hMCM subunits in the presence of DNA (Fig. 4*C*, *red circle*) could be either the bound 45-bp double-stranded portion of the DNA substrate (as ssDNA is too small to be clearly visualized at this resolution) or a protein domain displaced by the presence of the DNA, such as a flexible C-terminal domain. Further work is required to determine which hMCM subunit binds DNA under these conditions.

hMCM is an important factor in cell proliferation and therefore, by extension, cancer development. The ability to produce significant quantities of hMCM for analysis is an important step forward. Our biochemical findings show that the recombinant complex is active *in vitro*, and our structural studies show that its conformation is altered when bound to DNA. Our system enables the targeted manipulation of individual proteins within the hMCM complex, providing the potential to address in detail the important differences between individual subunits in the hMCM heterohexamer. It also provides the potential to develop screens for clinically relevant hMCM inhibitors.

## Footnotes

7 The abbreviations used are:

MCM: minichromosome maintenance
hMCM: human MCM.
